# Zinc Binding to Tau Influences Aggregation Kinetics and Oligomer Distribution

**DOI:** 10.3390/ijms20235979

**Published:** 2019-11-27

**Authors:** Guilherme G. Moreira, Joana S. Cristóvão, Vukosava M. Torres, Ana P. Carapeto, Mário S. Rodrigues, Isabelle Landrieu, Carlos Cordeiro, Cláudio M. Gomes

**Affiliations:** 1 Biosystems and Integrative Sciences Institute, Faculdade de Ciências, Universidade Lisboa, 1749-016 Lisbon, Portugal; ggmoreira@fc.ul.pt (G.G.M.); jmcristovao@fc.ul.pt (J.S.C.); apcarapeto@fc.ul.pt (A.P.C.); mmrodrigues@fc.ul.pt (M.S.R.); 2 Departamento de Química e Bioquímica, Faculdade de Ciências, Universidade Lisboa, 1749-016 Lisbon, Portugal; vmtorres@fc.ul.pt (V.M.T.); cacordeiro@fc.ul.pt (C.C.); 3 FTICR e Structural MS laboratory, Faculdade de Ciências, Universidade Lisboa, 1749-016 Lisbon, Portugal; 4 Departamento de Física, Faculdade de Ciências, Universidade Lisboa, 1749-016 Lisbon, Portugal; 5 Unité de Glycobiologie Structurale et Fonctionnelle, Université Lille, Centre National de la Recherche Scientifique, UMR 8576, F-59000 Lille, France; isabelle.landrieu@univ-lille.fr

**Keywords:** protein aggregation, Tau, intrinsically disordered protein, amyloid, neurodegeneration, zinc, calcium, spectroscopy, mass spectrometry

## Abstract

Metal ions are well known modulators of protein aggregation and are key players in Alzheimer’s Disease, being found to be associated to pathologic protein deposits in diseased brains. Therefore, understanding how metals influence amyloid aggregation is critical in establishing molecular mechanisms that underlie disease onset and progression. Here, we report data on the interaction of full-length human Tau protein with calcium and zinc ions, evidencing that Tau self-assembly is differently regulated, depending on the type of bound metal ion. We established that Tau binds 4 Zn^2+^ and 1 Ca^2+^ per monomer while using native mass spectrometry analysis, without inducing order or substantial conformational changes in the intrinsically disordered Tau, as determined by structural analysis using circular dichroism and Attenuated Total Reflectance-Fourier Transform Infrared (ATR-FTIR) spectroscopies. However, Tau aggregation is found to proceed differently in the calcium- and -zinc bound forms. While the rate of aggregation, as determined from thioflavin-T (ThT) fluorescence kinetics, is highly increased in both cases, the reaction proceeds via different mechanisms, as evidenced by the absence of the lag phase in the reaction of zinc-bound Tau. Monitoring Tau aggregation using native mass spectrometry indeed evidenced a distinct distribution of Tau conformers along the reaction, as confirmed by dynamic light scattering analysis. We propose that such differences arise from zinc binding at distinct locations within the Tau sequence that prompt both the rapid formation of seeding oligomers through interactions at high affinity sites within the repeat domains, as well as amorphous aggregation, through low affinity interactions with residues elsewhere in the sequence, including at the fuzzy coat domain.

## 1. Introduction

Alzheimer’s disease (AD) is the most common neurodegenerative disorder in the world and it has specific features, such as progressiveness and unremitting advancement, which result in a progressive decline of cognitive functions [1]. One common characteristic of this disease with most of other neurodegenerative diseases is the formation of amyloidogenic aggregates. In AD, the proteins that are involved in the pathogenesis are the amyloid-β (Aβ) peptide and Tau protein. The aggregation of these proteins culminates in the development of extracellular senile plaques, composed of Aβ peptides, and intracellular neurofibrillary tangles (NFTs), composed of the hyperphosphorylated Tau protein [2]. These species and/or the on-pathway species are cytotoxic and they lead to neuronal death [3]. In the past decades, there was great effort to alter AD pathology by targeting Aβ; however, this approach has been largely unsuccessful. Thus, interest in Tau-targeting strategies and studies is greatly increasing [4].

Metal ions have a major role in the physiological functioning of the brain and their homeostatic dysfunction is reported in several neurodegenerative diseases, including AD [5]. The main metal ions that are involved in neuronal physiological activity are iron (Fe^2+^/Fe^3+^), copper (Cu^2+^), zinc (Zn^2+^), and also calcium (Ca^2+^), whose homeostasis is also particularly important during aging [6,7]. All of these metal ions must be finely maintained under physiological levels, given their importance in neuronal physiology. However, in AD pathology, the observation of a significant imbalance in metal ion concentration and localization has prompted researchers to propose AD to also be considered a metallopathy [8]. In fact, with aging and AD progressiveness, there is an alteration in the localization and levels of neuronal metals and a marked accumulation within protein deposits. Moreover, the described cross-talk between the metal ions and AD pathological proteins suggests a close relationship between protein misfolding, aggregation, and metal ion homeostasis [9]. Zn^2+^ is particularly relevant in AD, since ex vivo observations indicate that senile plaques are highly enriched in this metal ion. Moreover, recent evidence indicates a complex interplay between Aβ and Zn^2+^ [10]. 

Several recent studies focused on the effect of zinc on Tau physiology and aggregation. The impact of zinc ions on Tau was investigated in the absence of heparin and it was found that zinc can induce a temperature-dependent reversible oligomerization of Tau [11]. However, these oligomers are amyloidogenic, and instantly dissociated following zinc chelation or temperature decrease. Moreover, isothermal titration calorimetry measurements have led to the hypothesis that zinc binds Tau to one high affinity binding site and three low affinity sites [11]. There are also findings that indicate that Zn^2+^ binds Tau and promotes its hyperphosphorylation through Zn^2+^-induced inactivation of protein phosphatase 2A [12] and the activation of Tau phosphorylation kinases [13]. Nevertheless, recent preclinical findings suggest a possible phosphorylation-independent pathway, in which Zn^2+^ might modulate Tau pathology, by direct-binding to Tau, and consequently promoting its aggregation [14]. Moreover, it was observed in in vitro studies that low concentrations of Zn^2+^ result in the acceleration of Tau fibril formation [15]. Additionally, it was demonstrated for Tau constructs and mutants with aggregation enhanced properties that, in the presence of zinc ions, Tau aggregation is further promoted with toxicity effects on neuroblastoma cell lines [16,17,18]. There are also studies that suggest what seems to be a tightly regulated Ca^2+^-dependent process of maintaining the state of Tau phosphorylation between physiological levels [19].

In the present study, we investigate the interaction of full-length human Tau with calcium and zinc ions while combining multiple biophysical and mass spectrometry approaches, providing evidence for multiple zinc binding sites, kinetic analysis, and changes in oligomer distribution. Our results lead to a conceptual model that accounts for the diverse reported effects of zinc binding to Tau aggregation.

## 2. Results

### 2.1. Native Mass Spectrometry Elicits Multiple Tau Species in the Presence of Calcium and Zinc 

**Figure 1:**
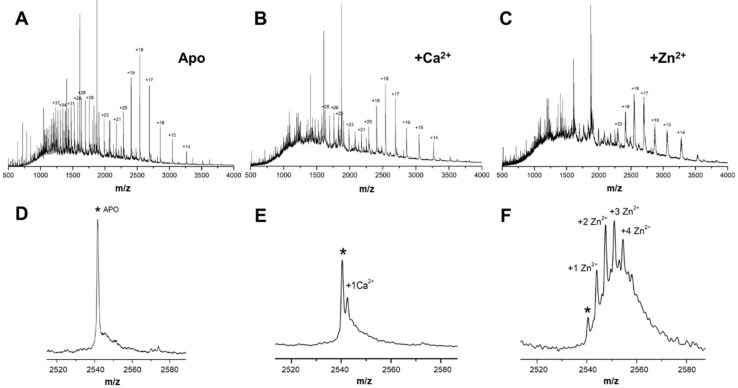
Native mass spectrometry analysis of Ca^2+^ and Zn^2+^ binding to hTau441. Native mass spectrum of hTau441 in the apo state (**A**) and in the presence of calcium (**B**) and zinc (**C**), with corresponding details of the +18 charge state multiplet for the apo (**D**), calcium (**E**), and zinc (**F**) bound states. Asterisks (*) denote the peak corresponding to apo (metal-free) hTau441. For assignments see Table S1.

Metal ion binding to Tau and its fragments has been previously reported, occurring in a range of stoichiometries and binding affinities. Here, we undertake a complementary approach in which native mass spectrometry (MS) is employed to analyze zinc and calcium binding to full length human Tau (hTau441), upon the incubation of purified protein with an excess of each of the metal ions, under native conditions. The native mass spectrum of hTau441 shows a broad distribution of charged states (Figure 1), from which a mass of 45,755 Da is inferred, in accordance with literature figures [20].

Analysis of the MS spectrum of metal free (apo) hTau441 reveals the presence of a single polypeptide with experimentally measured mass in excellent concordance with the molecular weight of the protein (Table S1). In the presence of Ca^2+^, low charged Tau protein species, most likely corresponding to more structured molecular forms, dominate the spectrum. Analysis of the multiplets in the +18 charge state evidence Tau with Ca^2+^ bound at a 1:1 stoichiometry, in equilibrium with apo protein (Figure 1E). A similar effect is observed in the presence of Zn^2+^, with a more pronounced shift to less charged protein species. Analysis of the multiplets of the +18 peak shows that, in this case, multiple zinc-bound Tau conformers are formed, with binding stoichiometries ranging from 1:1 to 4:1, being compatible with the occurrence of multiple Zn^2+^ binding sites in hTau441 (Figure 1F and Table S1). Similar analysis of apo hTau441 evidences a single +18 charge peak (Figure 1D).

Given the multiple Tau species that were observed by native MS in the presence of these metal ions, we posited that this might contribute to decreasing protein disorder. We have employed Far-UV circular dichroism (CD) and Attenuated Total Reflectance-Fourier Transform Infrared (ATR-FTIR) spectroscopies to compare secondary structure in the different conditions in order to determine whether calcium and zinc binding to hTau40 promoted some type of structural rearrangements on the polypeptide. Under the tested conditions, we obtained no evidence for substantial conformational changes in intrinsically disordered Tau, induced either by Ca^2+^ or by Zn^2+^ coordination (Figure S1).

### 2.2. Effects of Zinc and Calcium on Heparin-Induced Aggregation of Full Length Tau 

**Figure 2:**
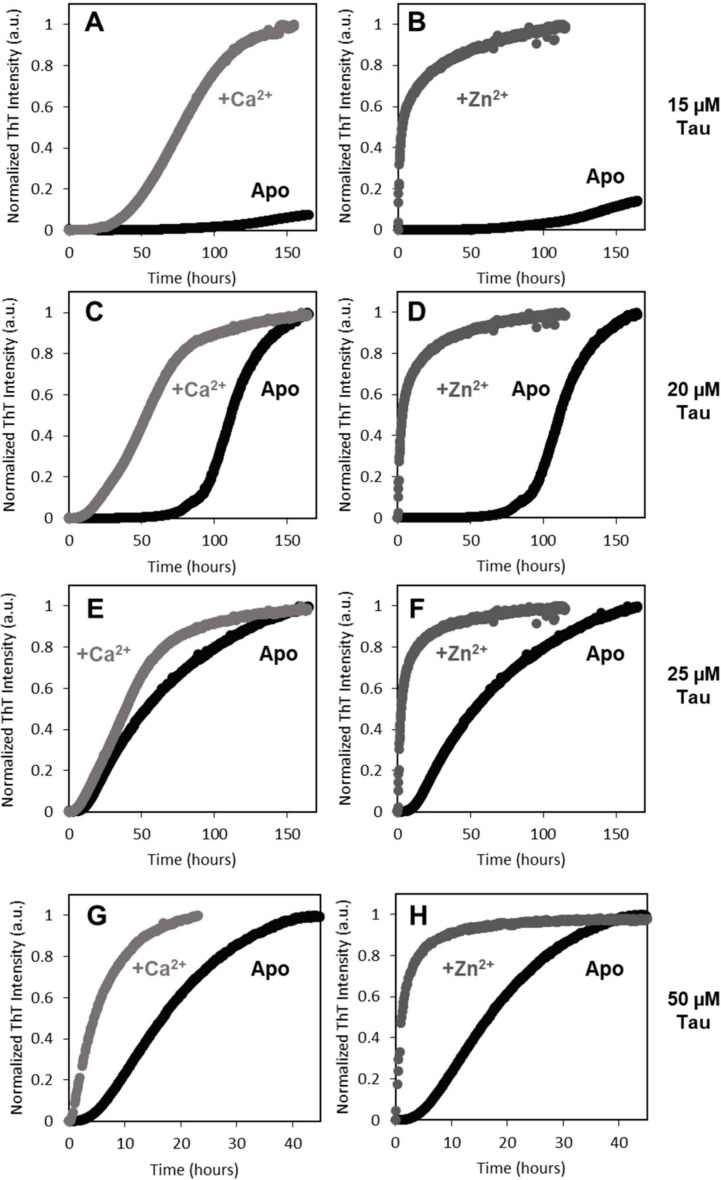
Metal ions promote aggregation of Tau. Tau aggregation in absence (black) or presence of 1.1 mM CaCl_2_ (light gray) or 1.1 mM ZnCl_2_ (dark grey), at increasing hTau441 concentrations: 15 µM (**A**,**B**), 20 µM (**C**,**D**), 25 µM (**E**,**F**) and 50 µM (**G**,**H**). All panels are representative of three independent measurements with Tau (at given concentration) at pH 7.4 with 1 mM DTT, 0.5 mg/mL heparin sodium salt, 50 mM NaCl, 1 mM PMSF and 75 µM ThT, under agitation (600 rpm) at 37 °C with or without metal ion. See the experimental section for further details.

Amyloid formation was monitored from thioflavin-T fluorescence in kinetic experiments, while using heparin as inducer of Tau aggregation, to establish the influence of zinc and calcium on the aggregation pathway of hTau441 [21,22]. In these experiments, we employed fractions that were enriched in monomeric Tau obtained through an optimized purification protocol, which resulted in highly reproducible kinetic traces, also across hTau441 preparations. The aggregation of Apo hTau441 proceeds via typical sigmoidal growth kinetics, as expected from a nucleated self-assembly reaction, whose rate increases as a function of the concentration of aggregating monomers. We carried out experiments covering a broad range of Tau concentrations (from 15 to 50 µM) in the absence and presence of calcium and zinc to test metal ion effects (Figure 2).

The aggregation rate of Tau is greatly enhanced by calcium- and -zinc binding, with the latter having a more prominent role, as inferred from effects on the lag phase (t_lag_) and reaction half time (Table 1). Across all of the tested hTau441 concentrations, zinc binding abolished the reaction lag phase, suggesting that, under these conditions, very fast nucleation processes occur that promptly trigger amyloid assembly. Calcium binding decreases the lag phase, which is still observed, and the reaction half time (t_½_) is consistently decreased across the tested range of Tau concentrations (Figure 2 and Table 1).

### 2.3. Analysis of Oligomer Distribution along the Aggregation Reaction

The distinct kinetic behavior of Tau aggregation that was observed under metal bound and metal free states suggests that distinct aggregation mechanisms predominate in the different conditions. To investigate this aspect, we proceeded with a characterization of the type, distribution, and dimensions of Tau aggregates that formed along the aggregation reaction in the apo, -calcium, and -zinc bound states. 

**Figure 3:**
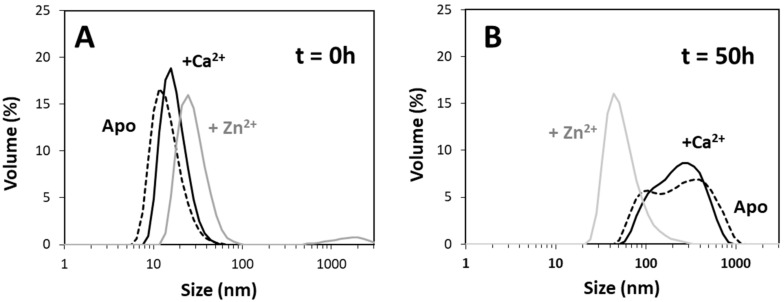
Dynamic light scattering analysis of soluble Tau oligomers. The distribution of particles in solution was analyzed in the beginning of heparin-induced aggregation of 50 µM Tau (**A**) and after 50 h at the plateau stage (**B**). Before each measurement samples were centrifuged (34,000× *g*, 10 min.) and supernatants of the different tested conditions analyzed: apo Tau (dotted line), in the presence of calcium (black line) and in the presence of zinc (grey line). Conditions for the aggregation reaction as those in Figure 1 (see materials and methods for details).

Dynamic light scattering (DLS) was employed to characterize the size distribution of Tau conformers immediately after the addition of calcium and zinc and after 50 h of incubation under aggregation conditions, which, in all cases, correspond to at least 85% of the maximum ThT emission intensity in kinetic experiments (Figure 3). Analysis of the conformers present at the beginning of the process evidences species with hydrodynamic radii of 11.7 nm (apo Tau), 15.7 nm (Ca^2+^-Tau), and 24.4 nm (Zn^2+^-Tau). The distribution of conformers is homogeneous in all cases, except for zinc-bound Tau, in which a small fraction (≈6%) of large aggregates (>100 nm) are observed (Figure 3A). This is compatible with the fast kinetics of Tau aggregation in the presence of Zn^2+^, and an indication that larger oligomers are forming within the preparation time of the sample for DLS analysis (<30 min.). 

**Figure 4:**
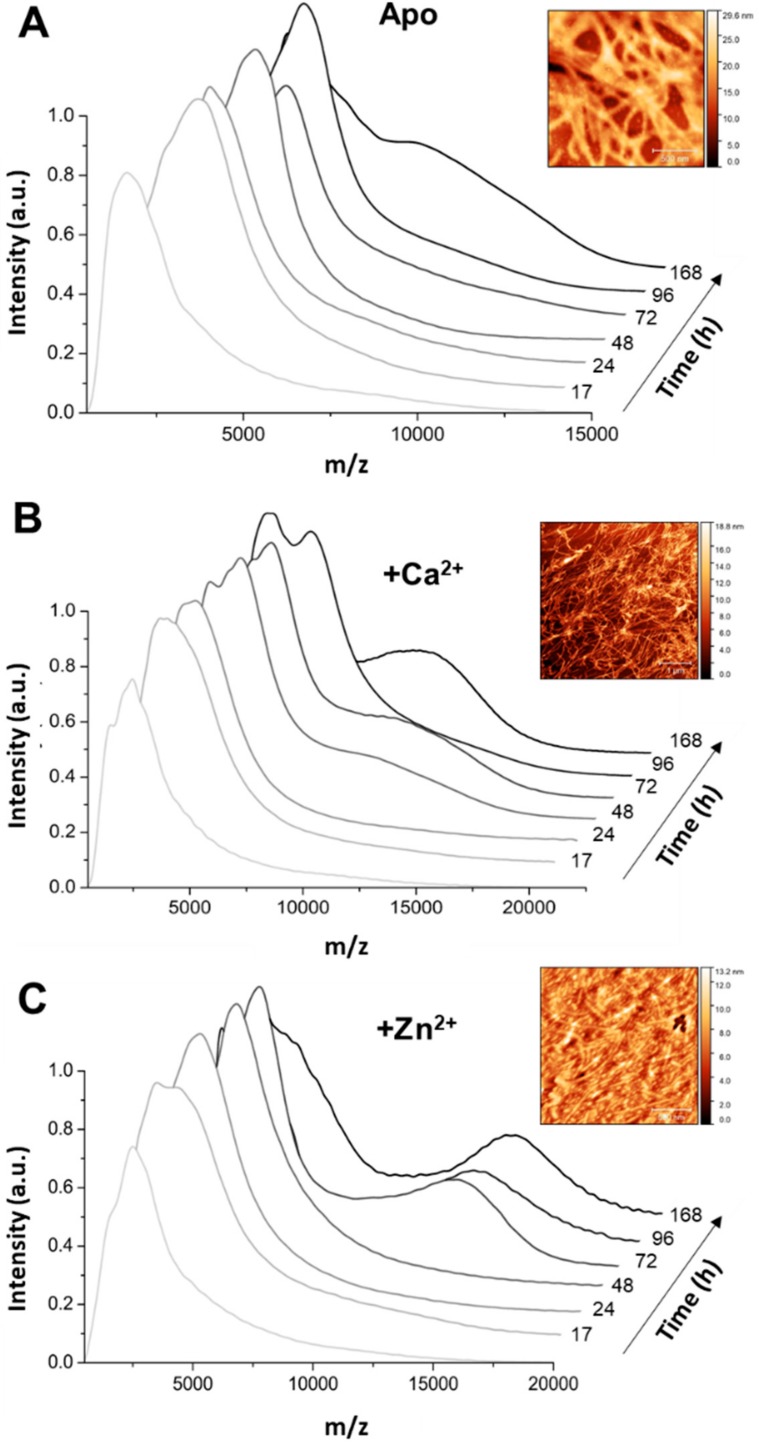
Different types of aggregates populate Tau aggregation time-points in different conditions. Native mass spectrum of Tau protein in near native state at pH 7.2 shows the formation of Tau aggregates during incubation at 37 °C with agitation (800 rpm). Samples were analysed at seven different time-points (0 h until 168 h corresponding from light grey to black). Aggregation of 35 µM Tau at pH 7.4 with 1 mM DTT, 0.5 mg/mL heparin sodium salt, 50 mM NaCl, 1 mM phenylmethanesulfonyl fluoride (PMSF) in the absence (**A**) and in presence of 1.1 mM CaCl_2_ (**B**) or ZnCl_2_ (**C**). Insets: Atomic Force Microscopy (AFM) images of Tau species at 50 h; scale bars: 500 nm in A and C, 1 μm in B.

Our data shows that the Ca^2+^-Tau, and Zn^2+^-Tau conformers have dimensions that are comparable to those of apo Tau. This finding agrees with the observation that metal ion binding is not inducing substantial conformational changes that result in secondary structure variations (Figure S1). We then carried out a similar analysis after 50 h of Tau aggregation, which evidenced interesting differences between the tested conditions (Figure 3B). The apo and calcium-bound Tau samples both exhibited a broad and heterogeneous distribution of large oligomers (100–400 nm); however, at this end point, the zinc-bound Tau aggregates were smaller (~43 nm) and relatively homogeneous. We further investigated this aspect by analyzing the distribution of Tau oligomers while using native mass spectrometry (Figure 4). 

Again, similar conformers were observed between apo-Tau and Ca^2+^-Tau, albeit aggregates formed faster in the presence of calcium (Figure 4A,B). However, the pattern of oligomers that formed during the aggregation of Zn^2+^-Tau is distinct, as the buildup of larger oligomers proceeds almost from the beginning of the reaction and a distinct distribution of aggregated species is present at the plateau phase of the kinetics (Figure 4C). Indeed, a comparison of the topography of Tau aggregates by Atomic Force Microscopy (AFM) in the presence of zinc is indicative of the persistence of shorter fibrils and oligomers in an amorphous protein layer background; on the other hand, longer mature fibrils are observed in the presence of calcium and in the apo form (Figure 4, insets). 

**Figure 5:**
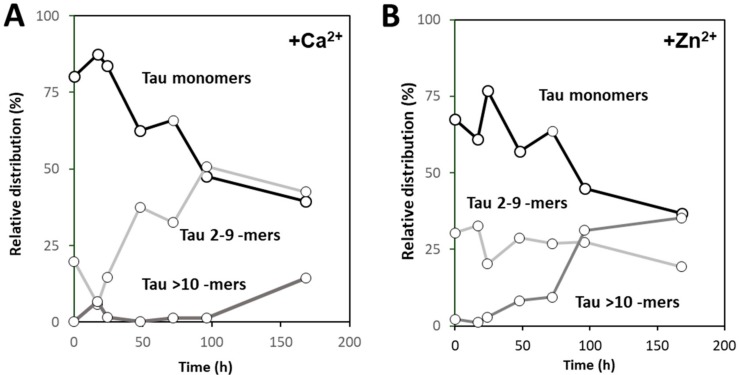
Influence of calcium (**A**) and zinc (**B**) on the distribution of Tau oligomers assessed by native mass spectroscopy. The distributions were obtained from peak fitting and deconvolution of the obtained spectra at each time point according to the relative contribution of hTau441 monomers, intermediate oligomers (Tau 2–9 -mers) and large oligomers (> Tau 10-mers).

We used native mass spectrometry to measure the distribution and evolution of selected oligomers over the reaction time to gain further insights into the type of oligomers forming during Tau aggregation (168 h). We performed peak fitting and deconvolution of the obtained spectra at each time point according to the relative contribution of hTau441 monomers, intermediate oligomers (Tau 2–9 -mers), and large oligomers (> Tau 10-mers) (Figure 5). Analysis of the Ca^2+^-Tau multimers evidences an interconversion of monomers into Tau 2–9 -mers up to 100 h of incubation, which corresponds to the onset of the plateau phase in the aggregation kinetics curves in Figure 1. From there on, larger aggregates start to build up, reaching ~15% of the overall species at the end point. In this case, the Tau monomers seem to undergo primary nucleation from which larger oligomers are formed (Figure 5A).

However, the scenario is distinct in the case of Zn^2+^-Tau multimers, as, from the very initial stages, the population of intermediate Tau oligomers is already ~30% of the total; in fact, these intermediate oligomers modestly decrease down to ~20% along the 168 h of incubation. The formation of larger Tau aggregates steadily increases up to 35% of total species along the reaction, with a concomitant decrease in Tau monomers (Figure 5B). This suggests that larger oligomers may be formed by secondary nucleation events that are mediated by surface catalyzed reactions of monomers with intermediate oligomers or other species in solution. In this context, the simultaneous formation of amorphous Tau aggregates via heparin independent processes [11] is also to be considered, as, in fact, the presence of zinc promotes the formation of visible light-scattering aggregates [11,16]. Therefore, it is plausible that organized self-assembly and amorphous aggregation of Tau simultaneously occur in the presence of zinc.

## 3. Discussion

Metal ion binding to aggregating proteins is a well-known regulatory mechanism of in vivo protein aggregation that is coupled to amyloid-forming age-related dementias. In the case of AD, it is established that metal dyshomeostasis is an altered cellular process that evolves along the pathology and neuronal protein inclusions in diseased human brains and in animal models accumulate metal ions, such as copper, iron, and zinc [23,24]. Several studies have focused on the effects of metal ion to binding Tau and its fragments, including zinc, while also advancing knowledge in the field; these studies have nevertheless opened questions that remain unaddressed [11,14,15,16,17,18]. Eventually, the understanding of the diverse consequences of zinc binding to Tau that has been reported to result in both stoichiometry-dependent effects on the kinetics of fibril formation and its cytotoxicity, as well as in amorphous aggregates, is the most interesting aspect to investigate. Here, contributed a new approach combining multiple biophysical and analytical methods, including native mass spectrometry, to study the effects of zinc and calcium interactions with full length human Tau on aggregation kinetics and the analysis of formed Tau conformers. 

Our investigations of zinc binding to Tau at pathological (µM) zinc concentrations, similar to those found upon traumatic brain injury and AD neurodegeneration [25], elicited multiple stable zinc binding sites that are, so far, unclear. We determined four Zn^2+^ binding sites in the protein while using monomer-enriched full-length human Tau and ESI-native mass spectrometry analysis (Figure 1). While binding of one zinc to Tau at micromolar affinities has been established in the R1/R3/R4-containing K19 construct and the R3 fragments [17], as well as in full length Tau [16] while using isothermal titration calorimetry, the possibility that Tau might accommodate multiple zinc binding sites has also been recognized [16]. Indeed, more recent work also based on calorimetric analysis of hTau441 titrated with zinc at high temperature has put forward the possibility that three low affinity and one high affinity zinc binding site exist in the protein [11]. However, the results reported here clearly show that such zinc binding moieties in Tau are also accessible at room temperature. The fact that we employed high resolution structural mass spectrometry here justifies the observation of multiple zinc binding sites, which have so far remained undetected by calorimetric methods at room temperature. Zinc binding to Tau has been previously assigned to Cys-291 and Cys-322 within the R2 repeat [15], and this is likely to be the major high-affinity zinc binding site. All Zn^2+^-Tau conformers present in solution are detected, thus providing solid evidence that Tau properties are amenable to regulation through binding of multiple zinc ions, since native MS approach does not discriminate between sites with distinct affinities or occupancies. A similar approach was undertaken to investigate calcium binding to Tau and it clearly evidenced a single binding site. Frequently Ca^2+^-binding sites in polypeptides result from the gathering of surface carbonyl and carboxylate centers that act as major donor groups. The identification of the sites for calcium binding sites within Tau is out of the scope of the present work, but the fact that, similarly to zinc, calcium also accelerates Tau aggregation suggests that its binding might also interfere with an association of Tau repeat regions. 

Indeed, calcium and zinc both strongly enhance Tau aggregation. As biophysical and spectroscopic analysis did not evidence metal-induced secondary structure changes and only induced mild differences on the protein hydrodynamic radius, the enhancement of aggregation must result from subtle conformational changes that promote aggregation. Indeed, previous work [17] has suggested that zinc binding to the R3 repeat of Tau weakens the aggregation-inhibitory interaction between R3 and R4, therefore resulting in faster Tau aggregation, and a similar mechanism might be in place for the Ca^2+^-Tau conformers. However, zinc has a much more dramatic effect on Tau rate. The aggregation of full length Zn^2+^-Tau proceeds promptly without lag phase, with the formation of aggregates visible at the naked eye; however, it should be noted that, in this study, we monitored the formation of amyloid fibrils using ThT fluorescence, unlike previous reports that followed the kinetics of hTau441 aggregation from turbidity measurements. We analyzed the type of particles in solution at the plateau stage of the aggregation reaction to investigate the morphology of the formed species. DLS analysis of soluble conformers (after centrifugation) after 50 h of incubation evidenced, in the case of apo and Ca^2+^-Tau, a broad distribution of species with dimensions in the 100–40 nm range, in agreement with the formation of large oligomeric species and fibrils. AFM characterization of total species indeed corroborates this analysis, as mostly fibrils are observed. However, in the case of Zn^2+^-Tau, a substantially different scenario emerged as only a predominant soluble species with a radius ≈44 nm was observed, which suggests the formation of smaller oligomers. AFM analysis of soluble and insoluble species evidence the existence of amorphous aggregates that coated the mica together with oligomers and a mixed population of shorter and longer fibrils. 

Using native MS and deconvolution of species along the reaction allowed for discriminating the different populations of oligomers evolving during the reaction, and establishing that zinc and calcium binding differently influence the aggregation pathway of hTau441. Calcium enhances aggregation by speeding the interconversion of monomers into intermediate aggregates (Tau 2–9 -mers), with the formation of large oligomer at the plateau stage of the ThT kinetics, corresponding to fibril formation, similarly to what is observed for apo hTau441. However, Zn^2+^-Tau evidences the instantaneous formation of intermediate aggregates (~30% of total species), which slightly decrease until ≈170 h incubation. Such species likely provide nuclei that promote the formation of larger oligomers at the expense of monomers, in secondary nucleation-like processes. 

**Figure 6:**
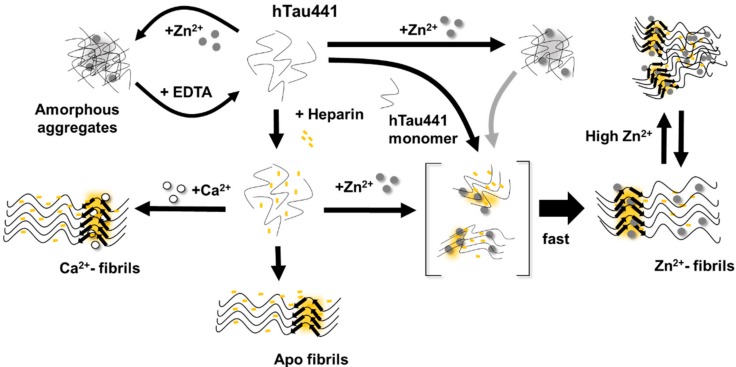
Schematic representation of the multiple conformers generated upon calcium and zinc binding to Tau. The presence of zinc enhances the heparin-dependent aggregation of hTau441 which very rapidly generates a distribution of toxic Thioflavin positive Tau aggregates and fibrils whose formation is also likely promoted by secondary nucleation events involving Tau monomers. Simultaneously, zinc binding to Tau in the absence of an aggregation enhancer promotes the reversible formation of amorphous aggregates, whose formation in the presence of heparin-induced aggregation cannot be excluded nor their involvement as aggregation seeds (grey arrow). It may be speculated that the latter would be favored by high zinc levels which might also promote the formation of amorphous aggregates in ThT-reactive species if low affinity zinc binding is taking place at moieties within the fuzzy coat regions; it is conceivable that these amorphous species are in equilibrium with ordered Zn^2+^-fibrils. Apo Tau fibrils are formed when hTau441 is incubated in the presence of heparin. See text for details and references.

All considered, we propose a general mechanism here, prompted by the observations reported, which reconciles the different findings of the effects of metals, and, in particular, zinc, in Tau aggregation (Figure 6). Zinc binding to residues within the R3/R4 regions are clearly responsible for faster ThT-reactive fibrillation. However, the effect of amorphous aggregations might result from lower affinity interactions of zinc to binding moieties elsewhere in the polypeptide. We speculate that this might involve binding of zinc to residues within the fuzzy coat regions, which contribute to the formation of unordered and amorphous aggregates, which are not observed in the presence of calcium, and that might also interfere with ordered stacking of residues in the repeat regions that will lead to amyloid cross-beta formation. Higher levels of zinc may promote some disorganization of fibrils, further contributing to sample heterogeneity. 

In conclusion, the binding of the two metals to Tau results in substantially different effects on the protein aggregation that open new clues into their implication in disease pathomechanisms. This is especially relevant when considering recent evidence implicating zinc binding to Tau to the generation of toxic aggregates, independently of phosphorylation [14]. 

## 4. Materials and Methods 

### 4.1. hTau441 Expression and Purification

All of the reagents were of the highest grade commercially available. ThT was obtained from Sigma. A Chelex resin (Bio-Rad) was used to remove contaminant trace metals from all solutions. The expression and purification of recombinant full-length human Tau (hTau441) was adapted from that described in [26]. *E. coli* (BL21 DE3) cells were transformed with the plasmid pET15b-Tau provided by I. Landrieu to obtain the overexpression of proteins (University of Lille, Lille, France), encoding the longest isoform of human Tau (441 amino acid residues). The transformed cells were plated in Luria-Bertani (LB) solid medium containing ampicillin (Nzytech, Lisbon, Portugal). A single colony was used to inoculate LB medium, being further incubated overnight at 37 °C. Bacterial cells were then grown in M9 medium and then induced with IPTG. The cells were harvested after 3h by centrifugation. The cell pellet was and resuspended in buffer A (50 mM Tris-HCl pH 6.5 and 1 mM EDTA (Sigma-Aldrich, St. Louis, MO, USA)), DNAse (PanReac, Applichem, Darmstadt, Germany), 1 mM phenylmethanesulfonyl fluoride (PMSF, Roth, Karsruhe, Germany), and freshly supplemented with complete™ EDTA-free protease inhibitor cocktail 1× (1 tablet, Roche). The bacterial cells were disrupted while using high-pressure French Press homogenizer at 20,000 psi, following centrifugation at 48,000× *g* and 4 °C for 1 h to remove insoluble materials. The bacterial cell extract was heated for 15 min. at 75 °C in a water bath and then centrifuged again to remove precipitated proteins. Cation-exchange chromatography (CEX) was performed in a Hiprep SP Fast-Flow 16/10 column (GE Healthcare, Chicago, IL, USA) while using a fast protein liquid chromatography (FPLC) ÄKTA purifier UPC 10 system (GE Healthcare, Chicago, IL, USA). Buffer A was used as running buffer and the protein of interested was eluted with buffer A containing 1 M NaCl. The fractions containing Tau were combined and the buffer was switched to buffer A. A protocol optimized for producing hTau441 monomers was developed, which consisted in urea (7.6 M) solubilization of post-chromatography fractions in the presence of 50 mM DTT, prior to passage on gel filtration while using a tricorn Superdex S-200 column. The eluted monomeric Tau samples were then lyophilized and stored at −20 °C. Tau purity was assessed by Mass spectrometry and SDS-PAGE, and the concentration was spectrophotometrically determined using $\varepsilon_{\mathrm{hTau}441}^{280\mathrm{nm}}$ = 7550 M^−1^ cm^−1^.

### 4.2. hTau441 Aggregation Kinetics

hTau441 aggregation kinetics were performed by recording the Thioflavin-T (ThT) fluorescence intensity as a function of time in a plate reader (FLUOstar OPTIMA, BMG Labtech, Ortenberg, Germany) with a 440 nm excitation filter and a 480 nm emission filter. The fluorescence was recorded while using bottom optics in half-area 96-well polyethylene glycol-coated black polystyrene microplates with a clear bottom (Corning, ref. 3881, New York, NY, USA). The microplates were sealed with transparent foil to avoid evaporation. The experimental concentrations of hTau441 varied between 15 to 50 µM. The concentration of heparin (heparin sodium salt from porcine) used was 0.5 mg/mL, DTT was 1 mM, NaCl was 50 mM [27], PMSF was 1 mM, ThT was 75 µM, and metal ions 1.1 mM (CaCl_2_ and ZnCl_2_). All of the samples were prepared while using 50 mM Tris-HCl pH 7.4. Tau aggregation was performed at 37 °C with an orbital shaking at 600 rpm for 300 s before each measurement.

### 4.3. Dynamic Light Scattering

DLS measurements were carried out in a Malvern Zetasizer Nano S instrument. hTau441 was analyzed from 50 μM samples in 50 mM Tris-HCl (pH 7.4) in the absence or presence of 1.1 mM CaCl_2_ or ZnCl_2_. The samples also contained the reagents at the same concentration than in the kinetics aggregation assays (except ThT). The reagents were filtered or centrifuged before use. Samples that were incubated at 37 °C and 800 rpm were analyzed at time points 0 h and 50 h. The samples were centrifuged for 10 min. at 34,000× *g* at room temperature and 50 µL aliquots from soluble fraction were collected and measured in a 45 µL quartz cuvette (Hellma) at 25 °C. The operating procedure was set to 17 runs, with each being averaged for eight measurements. The resulting data were analyzed while using DTS (version 7.01) software (Malvern, Worcestershire, UK).

### 4.4. Circular Dichroism

CD measurements were performed on a Jasco J-1500 spectropolarimeter equipped with a Peltier-controlled thermostated cell support. Far UV-CD spectra (200 to 260 nm) were recorded at 10 μM hTau441 with or without 40 μM CaCl_2_ or ZnCl_2_ in 50 mM Tris-HCl (pH 7.4). Individual solutions of Tau with and without metal ions were incubated for 1 h at 4 °C.

### 4.5. ATR-FTIR

Fourier-transform infrared spectroscopy was performed in a Bruker Tensor FTIR instrument that was equipped with an attenuated total reflection cell (Harrick) (ATR-FTIR) to assess the secondary structure of protein samples. The final volume analyzed was 20 µL with 4 µM hTau441. Metal ions, like CaCl_2_ and ZnCl_2_, were used at a final concentration of 16 µM. The samples were incubated at 4 °C for 1 h. The spectra were obtained from 4000 to 900 cm^−1^, with a 4 cm^−1^ resolution and 120 sample scans.

### 4.6. Native Mass Spectroscopy

The samples for mass spectrometry were delivered in concentration of 35 µM, and sample clean-up was performed while using Micro Bio-spin™ 6 columns (Bio-Rad, Hercules, CA, USA). The buffer used for native MS analysis was 100mM ammonium bicarbonate at pH 7.2. Mass spectrometry analysis was performed on a modified Waters QTOF Ultima II instrument with an off-line nano electrospray source. The samples were introduced into the QTOF mass analyzer while using in-house built capillary emitters (Sutter P-1000, BF120-69-10). Mass calibration was completed using 100 mg/mL of CsI in water in range of 1 to 20kDa. Data analysis of obtained mass spectra was accomplished while using MassLynx 4.1, Origin 8 Pro and PeakFIt v 4.12.

### 4.7. Atomic Force Microscopy

The topography/morphology of the samples was characterized by AFM with a PicoSPM LE (Molecular Imaging) system and Agilent Technologies PicoView 1.14.4 software (Keysight Technologies, Santa Rosa, CA, USA). The images were obtained in air, at room temperature, with HQ:NSC35/Hard/Al BS and HiRes-C14/Cr-Au µmasch cantilevers in dynamic mode. Approximately 10 µL of each Tau solution was deposited in freshly cleaved mica (Agar Scientific, Stansted, UK). The samples were allowed to rest for about 20 min. before rinsing with water and then dried in air before imaging.

## Figures and Tables

**Table 1:** Phase (t_lag_) and reaction half time (t_½_) of heparin-induced Tau aggregation (Figure 2).

[Tau] (µM)	Apo Tau		Ca^2+^-Tau	
	t_lag_ (h)	t_1/2_ (h)	t_lag_ (h)	t_1/2_ (h)
15	91.8	209	36.1	76.5
20	86.4	112	19.4	51.3
25	14.1	56.7	10.8	37.3
50	5.98	18.1	0.875	4.32

## Supplementary Materials

Supplementary materials can be found at https://www.mdpi.com/1422-0067/20/23/5979/s1.

## Abbreviations

AD	Alzheimer’s Disease
AFM	Atomic Force Microscopy
ATR	Attenuated Total Reflectance
Aβ	Amyloid-β Peptide
CD	Circular Dichroism
CEX	Cation-Exchange Chromatography
DLS	Dynamic Light Scattering
DTT	Dithiothreitol
FPLC	Fast Protein Liquid Chromatography
FTIR	Fourier-Transform Infrared Spectroscopy
hTau441	Full-length Human Tau Protein
LB	Luria-Bertani
MS	Mass Spectrometry
NFT	Neurofibrillary Tangles
PAGE	Polyacrylamide Gel Electrophoresis
PMSF	Phenylmethanesulfonyl Fluoride
T_½_	Half-time
ThT	Thioflavin T

## References

[1] Burns A., Iliffe S. (2009). Alzheimer’s disease. BMJ.

[2] Braak H., Braak E. (1997). Frequency of stages of Alzheimer-related lesions in different age categories. Neurobiol. aging.

[3] Andorfer C., Acker C.M., Kress Y., Hof P.R., Duff K., Davies P. (2005). Cell-cycle reentry and cell death in transgenic mice expressing nonmutant human tau isoforms. J. Neurosci..

[4] Congdon E.E., Sigurdsson E.M. (2018). Tau-targeting therapies for Alzheimer disease. Nat. Rev. Neurol..

[5] Leal S.S., Botelho H.M., Gomes C.M. (2012). Metal ions as modulators of protein conformation and misfolding in neurodegeneration. Coord. Chem. Rev..

[6] Leal S.S., Gomes C.M. (2015). Calcium dysregulation links ALS defective proteins and motor neuron selective vulnerability. Front. Cell. Neurosci..

[7] Egorova P., Popugaeva E., Bezprozvanny I. (2015). Disturbed calcium signaling in spinocerebellar ataxias and Alzheimer’s disease. Semin. Cell Dev. Biol..

[8] Barnham K.J., Bush A.I. (2014). Biological metals and metal-targeting compounds in major neurodegenerative diseases. Chem. Soc. Rev..

[9] Zatta P., Drago D., Bolognin S., Sensi S.L. (2009). Alzheimer’s disease, metal ions and metal homeostatic therapy. Trends Pharmacol. Sci..

[10] Lovell M.A., Robertson J.D., Teesdale W.J., Campbell J.L., Markesbery W.R. (1998). Copper, iron and zinc in Alzheimer’s disease senile plaques. J. Neurol. Sci..

[11] Roman A.Y., Devred F., Byrne D., La Rocca R., Ninkina N.N., Peyrot V., Tsvetkov P.O. (2019). Zinc Induces Temperature-Dependent Reversible Self-Assembly of Tau. J. Mol. Biol..

[12] Sun X.Y., Wei Y.P., Xiong Y., Wang X.C., Xie A.J., Wang X.L., Yang Y., Wang Q., Lu Y.M., Liu R. (2012). Synaptic released zinc promotes tau hyperphosphorylation by inhibition of protein phosphatase 2A (PP2A). J. Biol. Chem..

[13] Kim I., Park E.J., Seo J., Ko S.J., Lee J., Kim C.H. (2011). Zinc stimulates tau S214 phosphorylation by the activation of Raf/mitogen-activated protein kinase-kinase/extracellular signal-regulated kinase pathway. Neuroreport.

[14] Huang Y., Wu Z., Cao Y., Lang M., Lu B., Zhou B. (2014). Zinc binding directly regulates tau toxicity independent of tau hyperphosphorylation. Cell Rep..

[15] Mo Z.Y., Zhu Y.Z., Zhu H.L., Fan J.B., Chen J., Liang Y. (2009). Low micromolar zinc accelerates the fibrillization of human tau via bridging of Cys-291 and Cys-322. J. Biol. Chem..

[16] Hu J.Y., Zhang D.L., Liu X.L., Li X.S., Cheng X.Q., Chen J., Du H.N., Liang Y. (2017). Pathological concentration of zinc dramatically accelerates abnormal aggregation of full-length human Tau and thereby significantly increases Tau toxicity in neuronal cells. Biochim. Biophys. Acta Mol. Basis Dis..

[17] Jiji A.C., Arshad A., Dhanya S.R., Shabana P.S., Mehjubin C.K., Vijayan V. (2017). Zn(2^+^) Interrupts R4-R3 Association Leading to Accelerated Aggregation of Tau Protein. Chemistry.

[18] Kozina N., Mihaljevic Z., Loncar M.B., Mihalj M., Misir M., Radmilovic M.D., Justic H., Gajovic S., Seselja K., Bazina I. (2019). Impact of High Salt Diet on Cerebral Vascular Function and Stroke in *Tff3^−/−^*/C57BL/6N Knockout and WT (C57BL/6N) Control Mice. Int. J. Mol. Sci..

[19] Bojarski L., Herms J., Kuznicki J. (2008). Calcium dysregulation in Alzheimer’s disease. Neurochem. Int..

[20] Nshanian M., Lantz C., Wongkongkathep P., Schrader T., Klarner F.G., Blumke A., Despres C., Ehrmann M., Smet-Nocca C., Bitan G. (2019). Native Top-Down Mass Spectrometry and Ion Mobility Spectrometry of the Interaction of Tau Protein with a Molecular Tweezer Assembly Modulator. J. Am. Soc. Mass Spectrom..

[21] Sibille N., Sillen A., Leroy A., Wieruszeski J.M., Mulloy B., Landrieu I., Lippens G. (2006). Structural impact of heparin binding to full-length Tau as studied by NMR spectroscopy. Biochemistry.

[22] Goedert M., Jakes R., Spillantini M.G., Hasegawa M., Smith M.J., Crowther R.A. (1996). Assembly of microtubule-associated protein tau into Alzheimer-like filaments induced by sulphated glycosaminoglycans. Nature.

[23] Ayton S., Lei P., Bush A.I. (2015). Biometals and their therapeutic implications in Alzheimer’s disease. Neurotherapeutics.

[24] Cristovao J.S., Santos R., Gomes C.M. (2016). Metals and Neuronal Metal Binding Proteins Implicated in Alzheimer’s Disease. Oxid. Med. Cell. Longev..

[25] Sensi S.L., Granzotto A., Siotto M., Squitti R. (2018). Copper and Zinc Dysregulation in Alzheimer’s Disease. Trends Pharmacol. Sci..

[26] Danis C., Despres C., Bessa L.M., Malki I., Merzougui H., Huvent I., Qi H., Lippens G., Cantrelle F.X., Schneider R. (2016). Nuclear Magnetic Resonance Spectroscopy for the Identification of Multiple Phosphorylations of Intrinsically Disordered Proteins. J. Vis. Exp..

[27] Ramachandran G., Udgaonkar J.B. (2011). Understanding the kinetic roles of the inducer heparin and of rod-like protofibrils during amyloid fibril formation by Tau protein. J. Biol. Chem..

