# Supplementary material for: Zinc Binding to Tau Influences Aggregation Kinetics and Oligomer Distribution
Source: Int J Mol Sci. 2019 Nov 27;20(23):5979. doi: 10.3390/ijms20235979 (PMC6928861; doi:10.3390/ijms20235979)
Supplement: Supplementary file 1 [file ijms-20-05979-s001.pdf]

International Journal of
Molecular Sciences

MDPI

Supplementary materials

Table S1. Determination of calcium and zinc binding stoichiometries to hTau441 from the analysis of the +18 peak multiplets (shown in Figure 1).

	Molecular weight (Da)	Experimental molecular weight (Da)
Apo hTau441	45800	45755 (±0.002)
hTau441 + 1 Ca^{2+}	45840	45794 (±0.002)
hTau441 + 1 Zn^{2+}	45865	45819 (±0.001)
hTau441 + 2 Zn^{2+}	45930	45883 (±0.003)
hTau441 + 3 Zn^{2+}	45995	45947 (±0.002)
hTau441 + 4 Zn^{2+}	46060	46011 (±0.001)

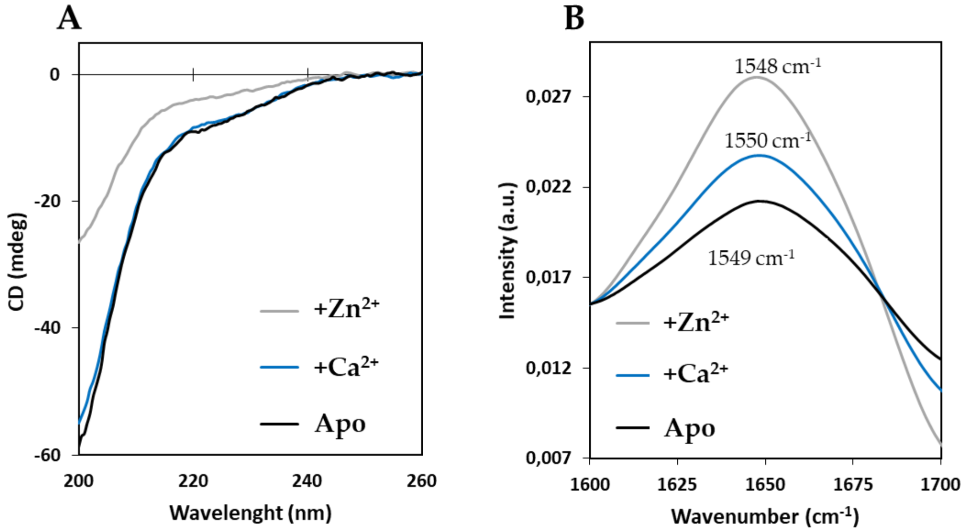

Figure S1. Metal ion binding to Tau does not change its structural conformation. (**A**) Circular dichroism spectra of 10 μM Tau in the absence (black) or in the presence of 40 μM $CaCl_2$ (blue) or 40μM $ZnCl_2$ (grey). (**B**) Attenuated total reflectance Fourier transform infrared spectra of 4 μM Tau in absence (black) or in presence of 16 μM $CaCl_2$ (blue) or 16 μM $ZnCl_2$ (grey). Measurements were performed after 1 h of incubation at 4 °C.
